# An integration of competing accounts on children’s number line estimation

**DOI:** 10.3389/fpsyg.2015.00884

**Published:** 2015-07-01

**Authors:** Tanja Dackermann, Stefan Huber, Julia Bahnmueller, Hans-Christoph Nuerk, Korbinian Moeller

**Affiliations:** ^1^Knowledge Media Research CenterTuebingen, Germany; ^2^Eberhard Karls University TuebingenTuebingen, Germany

**Keywords:** mental number line, number line estimation, model fitting, numerical development, spatial representation of number magnitude

## Abstract

Children’s estimation patterns in bounded number line estimation (NLE) reveal marked developmental changes. Three different theoretical accounts were proposed to explain these changes: a log-to-linear shift account, a proportion-judgment account and a two-linear account considering familiarity with numbers or the understanding of the place-value structure of the Arabic number system. However, only the first two accounts are considered prominently in the ongoing scientific debate. Therefore, we first present a reanalysis of NLE data of Austrian first-graders contrasting all three accounts. Results indicate that the two-linear account is a reliable alternative to the log-to-linear shift as well as the proportion-judgment account. However, we do not claim the two-liner account to provide an exhaustive explanation for the observed developmental changes. We rather introduce the idea that aspects of all three accounts may complement – instead of exclude – each other. Jointly considering conceptual (i.e., familiarity, place-value) and procedural (i.e., proportion-judgments) aspects will allow for a more comprehensive understanding of children’s development in NLE.

The mental number line (MNL) is a common metaphor to characterize the spatial representation of number magnitude. Based on converging behavioral and neuropsychological evidence (e.g., Fischer and Shaki, 2014 for a review), number magnitudes are assumed to be spatially organized along a left-to-right oriented MNL (see Göbel et al., 2011 for cultural influences). The bounded number line estimation (NLE) task is a task commonly used to draw inferences on children’s MNL representation (e.g., Siegler and Opfer, 2003; Siegler and Booth, 2004; Booth and Siegler, 2006; Moeller et al., 2009). The task requires participants to estimate the position of a target number (e.g., 34) on an empty number line with labeled endpoints (e.g., 0–100). Children of different ages were observed to perform differently on the same number range. Younger children tend to overestimate small numbers and compress larger numbers toward the end of the scale (e.g., first-graders on the 0–100 scale), whereas older children’s estimates on the same number range (e.g., third-graders) are spaced more accurately (e.g., Siegler and Booth, 2004).

Three distinct theoretical accounts were postulated to explain these developmental changes: (1) the log-to-linear shift account (e.g., Siegler and Opfer, 2003; Siegler and Booth, 2004; Booth and Siegler, 2006; Opfer and Siegler, 2007), (2) the proportion-judgment account (e.g., Barth and Paladino, 2011; Slusser et al., 2013), and (3) a two-linear account arguing for the influence of number familiarity (e.g., Ebersbach et al., 2008) or children’s understanding of the place-value structure (Moeller et al., 2009) to influence NLE performance. However, there is currently no study contrasting all three accounts. Yet, the latter seems particularly important for the ongoing debate on these competing accounts (e.g., Opfer et al., 2011; Slusser et al., 2013). In particular, contrasting all three accounts would allow for a more general explanation, for example, of inter-individual differences in estimation patterns observed in children of the same age or the impaired estimation performance of children with mathematical difficulties (e.g., Geary et al., 2012; Landerl, 2013). In turn, a more comprehensive understanding of the processes contributing to NLE performance may also help to narrow down the origin of the close association between NLE performance and numerical as well as mathematical competencies (e.g., Booth and Siegler, 2006, 2008; Laski and Siegler, 2007; but see LeFevre et al., 2013).

Therefore, we first present a reanalysis of NLE data of Austrian first-graders contrasting all three accounts before we discuss the idea that none of the accounts may provide an exhaustive explanation for the observed developmental aspects but that they may complement – instead of exclude – each other.

## Competing Accounts

Following the log-to-linear shift account (e.g., Siegler and Opfer, 2003; Siegler and Booth, 2004) children’s estimation patterns are supposed to directly reflect the spatial layout of the MNL representation: An underlying logarithmic representation was suggested to account for younger children’s tendency to overestimate small numbers. In contrast, the more accurately spaced estimation pattern of older children was interpreted to reflect an equidistantly linear underlying MNL representation. The respective log-to-linear shift was postulated to depend on age and number range (Booth and Siegler, 2006, 2008).

Questioning the idea of a representational shift, Barth and Paladino (2011; Slusser et al., 2013) argued that estimation performance rather reflects strategies applied to solve the bounded NLE task. They argued that the task always requires processing of a target number in relation to the given whole (i.e., the start- and endpoint of the scale). Corroborating their claim, Barth and Paladino (2011) found that seemingly linear estimation patterns were explained best by cyclic power models which indicate the use of reference points for estimation and thus proportion-based judgments (e.g., Hollands and Dyre, 2000). For example, one- and two-cycle power models (indicating the use of two or three reference points) were observed to provide the best fit for 7-year-old’s estimates on a 0-to-100 scale (Barth and Paladino, 2011). Following this proportion-judgment account, developmental changes in estimation patterns are associated with the increasing use of reference points (Barth and Paladino, 2011; Slusser et al., 2013).

This argument led to a controversial debate on whether there is indeed a representational shift or NLE performance simply reflects the application of specific estimation strategies (Barth and Paladino, 2011; Barth et al., 2011; Opfer et al., 2011; see also Ashcraft and Moore, 2012; Hurst et al., 2014). Importantly, however, there is a third account missing in this discussion. Ebersbach et al. (2008) and Moeller et al. (2009) suggested children’s NLE patterns to be accounted for best by two-linear regression models reflecting children’s familiarity with numbers or their place-value understanding, respectively (but see Muldoon et al., 2013, for diverging results). Ebersbach et al. (2008) found that estimation patterns of 5- to 9-year olds on a 0-to-100 scale were explained best by assuming two linear representations, one with a steeper slope for numbers familiar to children (as indicated by children being able to count to these numbers) and another one with a flatter slope for larger numbers not yet familiar to them. Thus, the breakpoint of the two linear segments reflects the upper end of the number range children were familiar with (see also Chesney and Matthews, 2013; Stapel et al., 2015).

Moeller et al. (2009; see also Helmreich et al., 2011; Moeller and Nuerk, 2011) proposed a similar two-linear account but with a fixed breakpoint at 10 considering children’s understanding of the place-value structure of the Arabic number system. Corroborating their claim, they observed that the two-linear model fitted first-graders’ estimation patterns on a 0-to-100 scale better than a logarithmic and a linear model. The authors argued that children’s overestimation of small numbers indicates children’s still insufficient understanding of linearity. This means that young children may not yet understand that the distance from 10 to 60 is exactly 10 times larger than the distance from 1 to 6 (Moeller et al., 2009). When this linear base-10 relation between single- and two-digit numbers may only be represented as “somewhat” but not 10 times larger, the two linear segments for single- and two-digit numbers differ in their slope with a steeper slope for single-digit numbers. With increasing age Moeller et al. (2009) propose children to become more proficient in integrating tens and units into a joint place-value structure which in turn leads the two segments to converge into a continuous linear one.

Taken together, each of the three theoretically differing accounts proposed to explain developmental changes in NLE was warranted by the better fit of the respective mathematical model. Yet, there is currently no study contrasting all three accounts while simultaneously considering critical aspects of model fittings (see Moeller and Nuerk, 2011; Opfer et al., 2011 for discussions).

## Mathematical Aspects of Model Fittings

To identify the best fitting model an important aspect regards the selection criteria indicating the goodness of model fit. Initially, *R*^2^ was used as indicator (Siegler and Opfer, 2003; Barth and Paladino, 2011). Taking into account different numbers of free parameters and, therefore, model complexity (e.g., two-linear vs. logarithmic functions), *adjusted R*^2^ (Moeller et al., 2009), the Akaike information criterion (*AIC or AIC_c_*, corrected for finite samples) and in recent studies also the Bayesian information criterion (*BIC;* e.g., Cohen and Sarnecka, 2014) served as indicators for goodness of fit. Additionally, Opfer et al. (2011; see also Slusser et al., 2013) argued for also considering generalizability of the models using cross validation. Cross validation is a model validation technique, in which the data are divided into a training and a test set. Models are fitted using the training set and their ability to generalize to new data is then determined by calculating the mean absolute percent error (MAPE) or the mean squared error (MSE) for the test set. Different models are then ranked according to their MAPE or MSE with smaller values indicating better model fits. A particular type of cross validation is leave one out cross validation where the test set consists of only one data point (LOOCV, see Browne, 2000, for more details; Opfer et al., 2011; Slusser et al., 2013, for application).

However, even if only one criterion is used to indicate the goodness of fit of the models tested, the most important question remains. How does one select the best fitting model? Descriptive analyses based on absolute values of model fitting parameters do not seem appropriate since model criteria often only show marginal differences (e.g., *R*^2^_model 1_ = 0.731; *R*^2^_model 2_ = 0.730; see Ebersbach et al., 2013). Similarly, using *t*-tests comparing *adj.R*^2^ values may not be adequate because it cannot be assumed that the parameters are normally distributed (Opfer et al., 2011; Ebersbach et al., 2013). Using a logit transformation might solve this problem (Baum, 2008). Similarly, logit transformation should work for other restricted values such as MAPEs. Because AIC and BIC values are obtained following the information-theoretic (Anderson, 2008) instead of the frequentist approach, there is no inferential statistic method to compare these. Instead of simply selecting the smallest AIC, Burnham and Anderson (2002) proposed to evaluate model fits according to Δ_i_ which describes the difference in AIC value with regard to the best fitting model (see Kass and Raftery, 1995, for a similar classification of ΔBIC). Burnham and Anderson (2002, p. 446; see also Slusser et al., 2013) proposed that “as a rough rule of thumb, models having Δ_i_ within 1–2 of the best model have substantial support and should receive consideration in making inferences. Models having Δ_i_ within about 4–7 of the best model have considerably less support, while models with Δ_i_ > 10 have either essentially no support and might be omitted from further consideration or at least fail to explain some substantial structural variation in the data.”

In sum, there are several criteria that seem appropriate to identify (the) best fitting model(s). However, in case more than one model fits well to the obtained data, it might be worthwhile to stop asking which model fits best and start asking why no clear ‘winner’ can be made out. In line with the recent discussion on log-to-linear shift vs. proportion-judgment, we will first evaluate which one of the associated models fits the data best.

## Which Model Fits Best?

We reanalyzed the data of Moeller et al. (2009) incorporating a sample of 128 Austrian first-graders (63 girls, mean age: 7 years, 4 months, SD = 7 months). Children were assessed at the end of first grade by 18 targets on the bounded NLE task on a 0-to-100 scale (i.e., 27, 2, 64, 35, 7, 13, 99, 75, 47, 3, 11, 82, 95, 9, 17, 6, 18, and 53). Additionally, 50 served as a practice trial. Children did not receive feedback about their performance.

**FIGURE 1:**
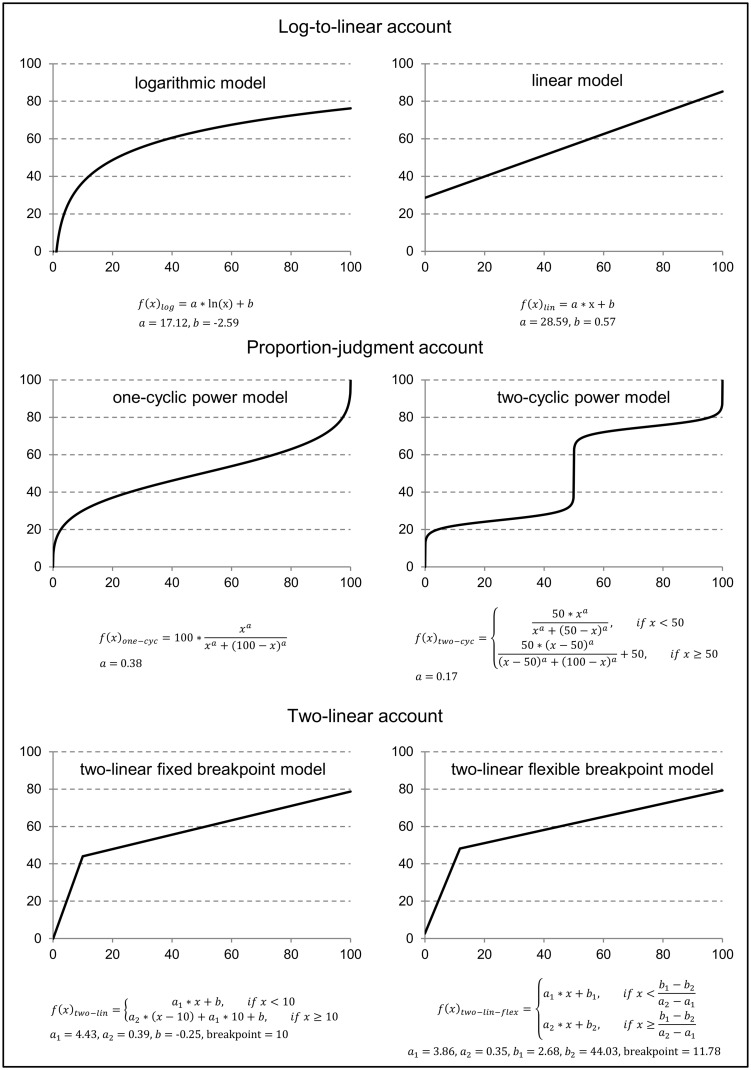
**Illustration of the three different accounts and their underlying model functions.** Lines depict model functions with mean parameter estimations of the current sample. Free parameters are a, a_1_, a_2_, b, b_1_, and b_2_.

Each mathematical model was fitted to the estimates of each child individually (see **Figure 1** for an illustration of the respective model functions including mean parameter estimates and breakpoints in case of the two-linear models). Linear and logarithmic models were fitted using two free parameters (i.e., the intercept and the slope); one- and two-cycle models were fitted with one free parameter (i.e., the exponent); the two-linear model with a fixed breakpoint at 10 was fitted with three free parameters (i.e., intercept and two different slopes for numbers <10 and for numbers >10, cf. Moeller et al., 2009) whereas the two-linear model with a flexible breakpoint was fitted with four free parameters (i.e., two intercepts and slopes for numbers smaller and larger than the breakpoint; cf. Ebersbach et al., 2008). Two children were excluded from analyses as their estimates only varied in the range between 47/49 and 55. MAPE and MSE values were obtained applying LOOCV method.

**Table 1** provides means of the different model selection criteria. Based on a descriptive account, the two-linear model with a fixed breakpoint at 10 seems to provide the best fit. Repeated measures ANOVA were computed on *n* = 107 or *n* = 117 children’s logit transformed *adj.R*^2^ and MAPEs. *Adj.R*^2^s for 19 children were negative and MAPEs for 9 children were >0.99 and could thus not be logit transformed. Furthermore, fittings of the two-cycle model were rather poor and *adj.R*^2^ mostly negative, which is why we excluded this model from further analyses. The ANOVA comparing logit transformed *adj.R*^2^ values revealed a significant effect of the factor model, *F*(4,106) = 35.80, *p* < 0.01, $\eta_{p}^{2}$ = 0.25. *Post hoc* Bonferroni-corrected pair-wise comparisons indicated a very consistent result pattern: We observed no difference between the two-linear models (*p* > 0.99) but both two-linear models provided a better model fit than did all other models (all *p* < 0.01). Furthermore, logit transformed *adj.R*^2^s of the other models also differed significantly (all *p* < 0.01). A similar pattern was observed for transformed MAPE values: The ANOVA revealed a significant main effect of the factor model, *F*(4,116) = 123.87, *p* < 0.01, $\eta_{p}^{2}$ = 0.52. *Post hoc* Bonferroni-corrected pair-wise comparisons indicated no significant difference between the two two-linear models (*p* > 0.99) but a significantly better fit of these two compared to all other models (*p* < 0.01). Except for the MAPEs of the logarithmic and one-cycle model (*p* = 0.08) all other MAPEs also differed significantly (*p* < 0.01).

**Table 1:** Means (across participants) of model fitting parameters for linear, logarithmic, one- and two-cycle functions as well as for two-linear functions with a fixed and flexible (flex) breakpoint.

	*Adj.R*^2^	AIC_c_	ΔAIC_c_	BIC	ΔBIC	MSE (LOOCV)	MAPE (LOOCV)
**Log-to-linear account**						
Linear	0.59	96.53	10.97	97.51	10.99	261.67	0.55
Logarithmic	0.69	92.99	7.43	93.97	7.45	205.17	0.43
**Proportion-judgment account**						
One-cycle	0.49	97.74	12.18	98.38	11.86	281.43	0.45
Two-cycle	0.04	108.58	23.02	109.22	22.70	554.19	0.50
**Two-linear account**						
Two-linear	**0.77**	**85.56**	**0.00**	**86.52**	**0.00**	**172.00**	**0.31**
Two-linear flex	**0.77**	89.90	4.34	90.39	3.87	172.94	**0.31**

*Bold face indicates best fitting model. ΔAIC_c_ and ΔBIC refer to the difference of AIC_c_/BIC values in reference to the model with the lowest AIC_c_/BIC; two-linear flex, two-linear model with a flexible breakpoint (cf. Ebersbach et al., 2008); AIC_c_, Akaike information criterion corrected for finite samples; BIC, Bayesian information criterion; MSE, mean squared error; LOOCV, leave one out cross validation; MAPE, mean absolute percent error.*

Regarding ΔAIC_c_ and also ΔBIC the logarithmic model is the only model that might be considered in further interpretations besides the two-linear models when applying the selection criteria suggested by Kass and Raftery (1995) or Burnham and Anderson (2002). According to both criteria, cyclic models and the simple linear model showed the worst fit to the data.

## The Two-Linear Account: A Plausible Alternative

Despite critical aspects of model fittings discussed above, these results highlight that the two-linear account, regardless of higher model complexity, is a reasonable approach to explain young children’s bounded NLEtwo-linear models fitted children’s estimates better than the models referring to other theoretical accounts. Interestingly, the breakpoints of the two two-linear models hardly differed (10 vs. 11.78). As it is implausible to assume that children are merely familiar with numbers up to 12 by the end of grade 1, this suggests that for the present data set both two-linear models represented place-value integration (see also Moeller et al., 2009). Importantly, these results corroborate findings of previous studies which argued bounded NLE to not directly reflect the MNL representation (e.g., Huber et al., 2014; Hurst et al., 2014). Thus, future debates on changes in children’s NLE performance should consider the two-linear account.

However, we do not claim the two-linear account to represent an exhaustive explanation of changes in children’s NLE performance. Instead, in the remainder of this article we discuss in how far the theoretically differing accounts might eventually complement each other to allow for a more comprehensive understanding of the development of children’s NLEs.

## A Comprehensive Approach on NLE

So far, investigating children’s spatial representation of number magnitude using the bounded NLE task was primarily characterized by trying to identify the best mathematical function to fit children’s estimates (Ebersbach et al., 2013 for an overview). Considering the diversity of methods across studies with respect to, e.g., time point of assessment, choice of targets, number line range, etc., we suggest an integration of the different theoretical aspects associated with the different mathematical models. In fact, the models may all capture specific aspects which are important for the development of children’s NLEs – but not necessarily at the same point in time. Following this rationale the three accounts may complement – instead of exclude – each other thereby providing a more comprehensive understanding of NLE. Thus, we propose an integrative account suggesting that children’s NLE patterns reflect different developmental stages of their understanding of multi-digit numbers (i.e., familiarity and place-value structuring) and proportional relations. Importantly, this integrative account is corroborated by recent data.

In line with previous studies, we assume NLE patterns to depend on the number range assessed and children’s age and experience with this range (cf. Siegler and Booth, 2004; Booth and Siegler, 2006). Initially, it seems that (young) children’s uncertainty with large magnitudes influences their estimation performance (Ebersbach et al., 2008; Stapel et al., 2015). By the end of grade 1 understanding of the place-value structure of the Arabic number system seems to influence estimation patterns most dominantly – at least in the number 0-to-100 (Moeller et al., 2009). This suggestion is corroborated by the current data indicating that children’s estimates assessed at the end of first grade are best fitted by the two-linear models both reflecting place-value integration. Nevertheless, both aspects seem to lead young children to perform direct estimates (as suggested by Siegler and Opfer, 2003; see also Slusser et al., 2013) – but in a biased way leading to an over-representation of the spatial extent of familiar and single-digit numbers, respectively. This results in estimation patterns that might look like a logarithmic layout but are theoretically and mathematically best explained by a two-linear account. However, the two-linear account has only been investigated within number ranges of 0-to-100 (as regards place-value understanding and familiarity) and 0-to-1000 (with respect to familiarity only; Ebersbach et al., 2008). The generalization of the two-linear account to other number ranges and the differentiation of influences of familiarity and place-value integration are still pending.

On the other hand, it seems reasonable to assume that with increasing age other aspects may become more prominent: Only after children are familiar with the respective number range and master the place-value structure of Arabic numbers, proportion-based estimation strategies are most beneficial. Thus, the proportion-judgment account of Barth et al. (2011) seems more appropriate for (older) children who are already confident with the respective number ranges and can thus start to use reference points for deriving their estimates. Application of such strategies then results in an estimation pattern best described by a one- or two-cycle model although estimates seem to follow a linear layout (cf. Barth and Paladino, 2011). In sum, we suggest that familiarity with a given number range as well as understanding the place-value structure allow for the application of solution strategies other than numerical estimation, such as proportion-judgments, in NLE.

This idea of the differing accounts complementing – instead of excluding – each other may also be relevant for the question about the origin of the association between NLE performance and basic numerical and arithmetic abilities. Recently, Link et al. (2014) provided evidence suggesting that it is highly unlikely that an underlying MNL representation causes this association. The authors investigated estimation performance in a similar, but unbounded NLE task (with only the start point and a unit, but no endpoint given) and found no correlation between estimation performance and either basic numerical or arithmetic competencies. Furthermore, Link et al. (2014) did not observe evidence for the application of proportion-based strategies in unbounded NLE (see also Cohen and Sarnecka, 2014). From this the authors concluded that basic numerical as well as arithmetic processes are needed to apply proportion-based strategies (such as calculating reference points, deciding whether the target number is smaller or larger than a chosen reference point, computing the difference from the reference point and the target, etc.). In turn, this drives the association of bounded NLE and basic numerical as well as arithmetic competencies. Importantly, these subordinate processes require familiarity with the given number range and an understanding of the place-value structure of the Arabic number system – and thus an integration of the processes suggested by the different accounts to explain for changes in children’s NLEs.

Taken together, we argue that estimation performance in the bounded NLE task is explained best by jointly considering children’s conceptual (i.e., familiarity, place-value understanding) and procedural (i.e., proportion-judgment) numerical knowledge and to a lesser extent by the nature of spatial-numerical representations *per se*. In turn, focusing on individual procedural and conceptual knowledge might allow for a better understanding what drives the reliable association of NLE performance and other numerical competencies. In this vein, it seems desirable for future studies to consider all available models (e.g., in terms of parameter estimates) and not only undifferentiated performance measures (such as estimation errors).

## Conflict of Interest Statement

The authors declare that the research was conducted in the absence of any commercial or financial relationships that could be construed as a potential conflict of interest.
